# Supplementary material for: Siderophores as an iron source for picocyanobacteria in deep chlorophyll maximum layers of the oligotrophic ocean
Source: ISME J. 2022 Mar 3;16(6):1636–46. doi: 10.1038/s41396-022-01215-w (PMC9122953; doi:10.1038/s41396-022-01215-w)
Supplement: Supplementary file 1 — Supplemental material [file 41396_2022_1215_MOESM1_ESM.pdf]

Supporting information for:

Siderophores as an iron source for picocyanobacteria in deep chlorophyll maximum layers of the oligotrophic ocean

Shane L. Hogle[1,2,*], Thomas Hackl[1,3], Randelle M. Bundy[4], Jiwoon Park[4], Brandon Satinsky[1], Teppo Hiltunen[2], Steven Biller[1,5], Paul M. Berube[1], Sallie W. Chisholm[1,6*]

[1]Massachusetts Institute of Technology, Dept. of Civil and Environmental Engineering, Cambridge, MA, USA
[2]Department of Biology, University of Turku, Turku, Finland
[3]Max Planck Institute for Medical Research, Biomolecular Mechanisms, Heidelberg, Germany
[4]School of Oceanography, University of Washington, Seattle, WA, USA
[5]Wellesley College, Biological Sciences, Wellesley, MA
[6]Massachusetts Institute of Technology, Dept. of Biology, Cambridge, MA, USA

[*]**Corresponding Authors:** Shane Hogle (shane.hogle@utu.fi) and Sallie W. Chisholm (chisholm@mit.edu)

This PDF file includes:
Supplementary Text
Supplementary Figures S1 to S6
Supplementary Tables S1 and S2

Contents

1 Supplementary Methods **3**

1.1 Data sources . . . 3

1.2 Preprocessing biogeochemical and hydrographic measurements . . . 3

1.3 Estimation of siderophore transport cluster prevalence in genomes . . . 4

1.4 Quality control of metagenome sequencing reads . . . 4

1.5 Metagenome read classification . . . 5

1.6 Processing and analysis of metagenomic count data . . . 5

1.7 Random Forest training and testing . . . 5

1.8 Seasonal effects from Generalized Additive Models . . . 6

1.9 Dataset and code availability . . . 6

List of Supplementary Figures

S1 Gene diagrams of siderophore transport clusters in marine picocyanobacteria . . . 7

S2 Comparing the sequence similarity of picocyanobacterial TonB dependent receptor (TBDR) genes . . . 8

S3 Biogeography of *Synechococcus* siderophore consumers . . . 9

S4 Seasonal dynamics of *Prochlorococcus* siderophore consumers in the N. Pacific (Station ALOHA) and N. Atlantic (Station BATS) . . . 10

S5 dFe concentrations from the N. Pacific (Station ALOHA) and N. Atlantic (Station BATS) from prior studies. . . . 11

S6 Other predictive variables of *Prochlorococcus* siderophore consumer abundance . . . 12

List of Supplementary Tables

S1 Regression of dFe, Fe-binding ligands, and siderophores at ALOHA and BATS . . . 13

S2 Beta regression of *Prochlorococcus* siderophore receptor abundance . . . 14

1 Supplementary Methods

1.1 Data sources

The collection of genomes searched in this study (MARMICRODB, https://zenodo.org/record/3520509) was previously introduced as a reference database for quantifying *Prochlorococcus* and SAR11 abundance in marine metagenomes [1]. These genomes were selected to provide a comprehensive representation of the marine environment and a nonredundant and simplified characterization of terrestrial and host-associated systems. MARMICRODB includes 18758 bacterial and archaeal single cell, metagenome-assembled, and isolate genomes and eukaryotic transcriptomes from a variety of sources [2–11] as well as over 7000 viral genomes from NCBI RefSeq [12].

The metagenomic collection consisted of 195 surface and deep chlorophyll maximum (DCM) metagenomes from Tara Oceans project [13] and 480 metagenomes acquired from GEOTRACES cruises [14]. *Prochlorococcus* clade abundances from GEOTRACES metagenomes are from a previous study [1]. *Prochlorococcus* clade cell concentrations from the Hawai'i Ocean Time-series Station ALOHA and the Bermuda-Atlantic Time-series Study BATS site were accessed from the Biological and Chemical Oceanography Data Management Office (https://www.bco-dmo.org/dataset/3381). Clades were measured by quantitative PCR as described in a prior study [15].

Trace metal and other chemical concentrations were obtained from the GEOTRACES Intermediate Data Product IDP2017 version 2 (accessed January 2019) [16]. Samples were included from sections GA02 [17, 18], GA03, GA10 [19], and GP13, which all had paired metagenomic data. Biogeochemical data from the Tara Oceans project was downloaded from https://doi.pangaea.de/10.1594/PANGAEA.875579. Climatological mean dissolved iron from the MIT Darwin model (v0.1_llc90, http://darwinproject.mit.edu/) was obtained using pycmap v0.1.2 [20]. This model version is modified from earlier versions [21, 22] and includes biogeochemical cycling of nutrient elements and a planktonic marine ecosystem model incorporating functional and size diversity with multiple links between trophic levels. It is driven by the physical ocean circulation model from ECCO v4 [1]. The iron cycle parameterization in the MIT Darwin model includes a dominant iron source through aeolian dust deposition and a dominant sink due to particle scavenging and dissolution/complexation with organic ligands. The model includes two chemical species - Fe' (inorganic free ferric iron) and FeL (organically complexed Fe). The total dissolved iron concentration consists of the sum of these two species.

1.2 Preprocessing biogeochemical and hydrographic measurements

Raw fluorescence or derived chlorophyll *a* concentrations from hydrographic CTD casts on the GEOTRACES cruises were obtained from the IDP17 [16]. At each sampling station, the fluorescence signal from the CTD profile was smoothed using a 10-meter rolling mean, then scaled at each depth by the profile standard deviation, and finally divided by the maximum value to obtain a normalized and scaled fluorescence profile from each sampling location. Normalization was necessary because fluorometer data from across the datasets was either in uncalibrated factory units, derived chlorophyll *a* concentrations, or raw signal and individual instrument calibration coefficients were unavailable. This normalization procedure discards the fluorescence signal magnitude, but it allows for a between-sample comparison of fluorescence profile shape and DCM depth. The DCM depth was defined as depths with fluorescence values within 90% of the peak subsurface fluorescence signal and contiguous to the maximum fluorescence depth. The full DCM depth range was defined as 50% of maximal subsurface fluorescence.

When metagenome bottle samples did not align precisely with the depth/coordinates of the hydrographic casts or trace metal casts, the nearest CTD cast (within a 100 km radius) was matched to discrete bottle

data from within a 15-meter depth range. Coordinates were matched using the shortest distance between two geospatial points according to the Haversine formula. Despite this relaxed, fuzzy-matching approach, some combinations of variables were missing for some observations in the dataset. Generally, features like salinity, temperature, and macronutrient concentrations were present for most metagenome observations. However, in fewer than 5% of all samples, we needed to impute at least one hydrographic parameter or macronutrient variable. We imputed missing salinity, temperature, macronutrient, and DCM depth observations by the missForest algorithm [23] using chaining random forest prediction and imputation from all other available variables except siderophore receptor abundance. This process was implemented with the ranger package [24] in missRanger v2.1.3 (`https://github.com/mayer79/missRanger`). The GEOTRACES trace metal data was not as complete as the macronutrient and hydrographic data. For example, measured dissolved iron or copper concentrations from GEOTRACES IDP17 were only available for roughly half of the total metagenome observations. Therefore, we also used random forest imputation for trace metal measurements, but we only imputed trace metal categories with no less than 50% missing measurements. Missing trace metal observations were imputed separately for each ocean basin (N. Atlantic, S. Atlantic, N. Pacific, S Pacific, Mediterranean and Red Seas, and Indian Ocean). The imputation workflow is documented at `https://github.com/slhogle/cyano-sidero-ocean`.

1.3 Estimation of siderophore transport cluster prevalence in genomes

Many of the analyzed *Prochlorococcus* and *Synechococcus* genomes were incomplete because they were sequenced from material isolated from single cells. Therefore, genome completeness was taken into account when estimating the proportion of genomes with the siderophore transport cluster. It was assumed that there was no bias in the content of genome recovery in the single-cell genome sequencing and assembly processes, which is reasonable for the latest single-cell technologies [25]. The number of missing base pairs in each incomplete assembly was estimated with checkM using a custom set of 730 single-copy, core genes from *Prochlorococcus* and *Synechococcus* genomes (`https://doi.org/10.5281/zenodo.3719132`). The corrected prevalence of the TonB dependent receptor (TBDR) gene (f) was estimated as:

$$f = \frac{\sum_{i=1}^{n} p_i}{\sum_{i=1}^{n} g_i} \times \sum_{i=1}^{n} \left(g_i / c_i\right) / \bar{p} / n$$

where for each taxonomic group/clade, p is the length of the TBDR gene in base pairs, g is the length of the genome assembly in base pairs, c is the completeness estimate, $\bar{p}$ is the average length of all TBDR genes from each taxonomic group/clade, and n is the total number of assemblies from each taxonomic group/clade. The TBDR gene always co-occurred with other transport components (ABC ATPase, ABC permease, ABC solute binding protein, TonB, ExbB, ExbD) as an operon-like structure within the picocyanobacterial genomes. Hence, it was assumed that the corrected prevalence of this individual gene represents that of the entire siderophore transport cluster.

1.4 Quality control of metagenome sequencing reads

Raw metagenomic reads were preprocessed using the bbtools software suite (`https://jgi.doe.gov/data-and-tools/bbtools/`) as described in detail earlier [1]. Briefly, reads were trimmed of sequencing adapters using kmer matches to Illumina adapter sequences from the 3' end of the read. A maximum Hamming distance of one was required for matches to reference kmers. Reads with three or more 'Ns' or with an average quality score of less than Q20 were discarded. Overlapping reads were trimmed based on insert size (tbo=t) if adapter kmers were not identified and then both

reads trimmed to the minimum length of the read pair. Reads shorter than 60 bp after all adapter trimming steps were discarded. Reads were also discarded if they contained common Illumina sequencing artifacts (based on kmer matches, k=31). Reads were overlapped using the bbmerge tool [26] using default parameters with a minimum insert size of 35 bp and a minimum overlapping sequence of 12 bp. All unmerged and orphaned reads that passed quality control were also retained for downstream annotation and analysis.

1.5 Metagenome read classification

Metagenome reads were mapped to MARMICRODB using Kaiju v1.6.0 [27] as described before [1]. Briefly, Kaiju was run in greedy mode, tolerating a maximum of five amino acid mismatches per match, a minimum match length of 11, a minimum BLOSUM62 score of 65, and excluded alignments with a Kaiju "expect value" (analogous to BLAST e value) less than 0.05. Query sequences with low-complexity regions were filutered using the SEG filtering algorithm from the BLAST+ package [28]. The majority of reads (54% ± 10%) were classified to at least one reference database sequence.

1.6 Processing and analysis of metagenomic count data

For each sample, reads with the best match to any *Prochlorococcus* or *Synechococcus* TonB dependent receptor (TBDR) gene were summed then length-normalized (reads per kilobase; RPK) to the median length of all *Prochlorococcus* or *Synechococcus* TBDR genes. A collection of 730 single-copy core genes (COGs) (`https://doi.org/10.5281/zenodo.3719132`) was used to estimate the abundance of *Prochlorococcus* and *Synechococcus* genomes in each sample. For each sample, reads mapping to each core COG were length-normalized to the median length of the COG. Samples with median *Prochlorococcus* or *Synechococcus* core COG coverage was less than 100 RPK were excluded. Outlier core COGs were excluded if they were not present in all samples and were outside the 10th and 90th RPK percentiles of all COGs across all samples. The outlier removal process resulted in 277 *Prochlorococcus* and 186 *Synechococcus* core COGs that were robust and stable across all samples (i.e., had no significant differences between ocean basins or depth ranges). The abundance of *Prochlorococcus* or *Synechococcus* genomes in each sample was taken as the median of the 277 or 186 length-normalized core COG abundances. The frequency of siderophore consumers was calculated as the ratio of length-normalized TBDR abundance to the abundance of *Prochlorococcus* or *Synechococcus* genomes (i.e., median COG abundances). Assuming that TBDRs are single-copy per genome, this value should approximate the proportion of *Prochlorococcus* or *Synechococcus* genomes in a metagenomic sample with the potential to consume siderophores.

1.7 Random Forest training and testing

A random forest regression was trained to predict the frequency of *Prochlorococcus* siderophore consumers using 45 different environmental features as input. These environmental features included trace metal data from GEOTRACES, macronutrient data from GEOTRACES and Tara Oceans, modeled climatological dFe means from the MIT Darwin model (`http://darwinproject.mit.edu/`), frequencies of different *Prochlorococcus* ecotypes, and water column chlorophyll a profiles. Samples were restricted to the upper 300 meters of the water column and where the median RPK of marker genes was higher than 100. Random forest hyperparameters were tuned using a grid of potential parameter combinations. Model training was performed using nested ten-fold cross-validation, reserving 20% of the data for estimating final model performance. Specifically, the number of variables to split at each tree node (mtry), the minimum number of observations to partition at each node (min.node.size), and the

forest size (ntrees) were tuned. Hyperparameters were selected by maximizing the coefficient of determination from correlation (model R^2) and minimizing the root mean square error (RMSE). Performance metrics were averaged over the ten cross-validation splits of the training data.

Random forest regression internally ranks predictor variables by their relative importance for completing a given prediction task, but it can not by itself assess whether the influence of a variable is greater than expected by chance. The latter task is necessary, for example, if one is interested in understanding the mechanisms governing a system rather than the simple construction of a "black-box" predictive tool. The Boruta heuristic was used to assess variable importance with 100 random permutations for feature selection and importance ranking [29]. Briefly, Boruta is designed to identify all dataset features more relevant to a random forest regression task than by chance alone. In practice, Boruta will identify variables that may be redundant/correlated or that weakly interact with other features to produce a correlation with the outcome variable greater than expected by chance. During one algorithm iteration, Boruta doubles each feature in a dataset, randomly shuffles the doubled variables' observed values, and constructs a random forest model with the original and randomized features. Then by using the precomputed importance rankings from the model construction step, Bortua compares the importance of the 'true' features to the maximum importance of the randomly shuffled 'shadow' features using a two-sided test of equality (p value cutoff of 0.01). This process is then repeated 100 times or until all features have a consistent designation of importance. By iterating this process, the algorithm identifies dataset features that consistently underperform or overperform relative to the randomized input variables.

1.8 Seasonal effects from Generalized Additive Models

Seasonal effects in time-series metagenomes were estimated using Generalized Additive Mixed Models and Linear Mixed-Effect Models implemented through the mgcv v1.8-26 [30] nlme v3.1-148 libraries in R v3.6.2. To decompose seasonal effects a cyclic spline term was fit to a numerical variable for "day of the year", which we assume is representative of the season. A global trend term was also fit to the cumulative time since sampling onset. Residuals are modeled as a time series process with a continuous AR(1) correlation structure to account for lack of residual independence. Seasonal effects were considered significant if the "day of the year" smooth term was statistically significant ($p < 0.05$). Fig. 2B shows the estimated smooth term for "day of the year" with 95% confidence intervals shaded.

1.9 Dataset and code availability

The data and code for generating all figures and tables and supporting the text's conclusions are available from `https://github.com/slhogle/cyano-sidero-ocean`.

The MARMICRODB dataset, including a comprehensive description, raw protein sequence files, Kaiju formatted databases, scripts, and instructions for using the resource, is available from `https://doi.org/10.5281/zenodo.3520509`.

Supplementary Figures:

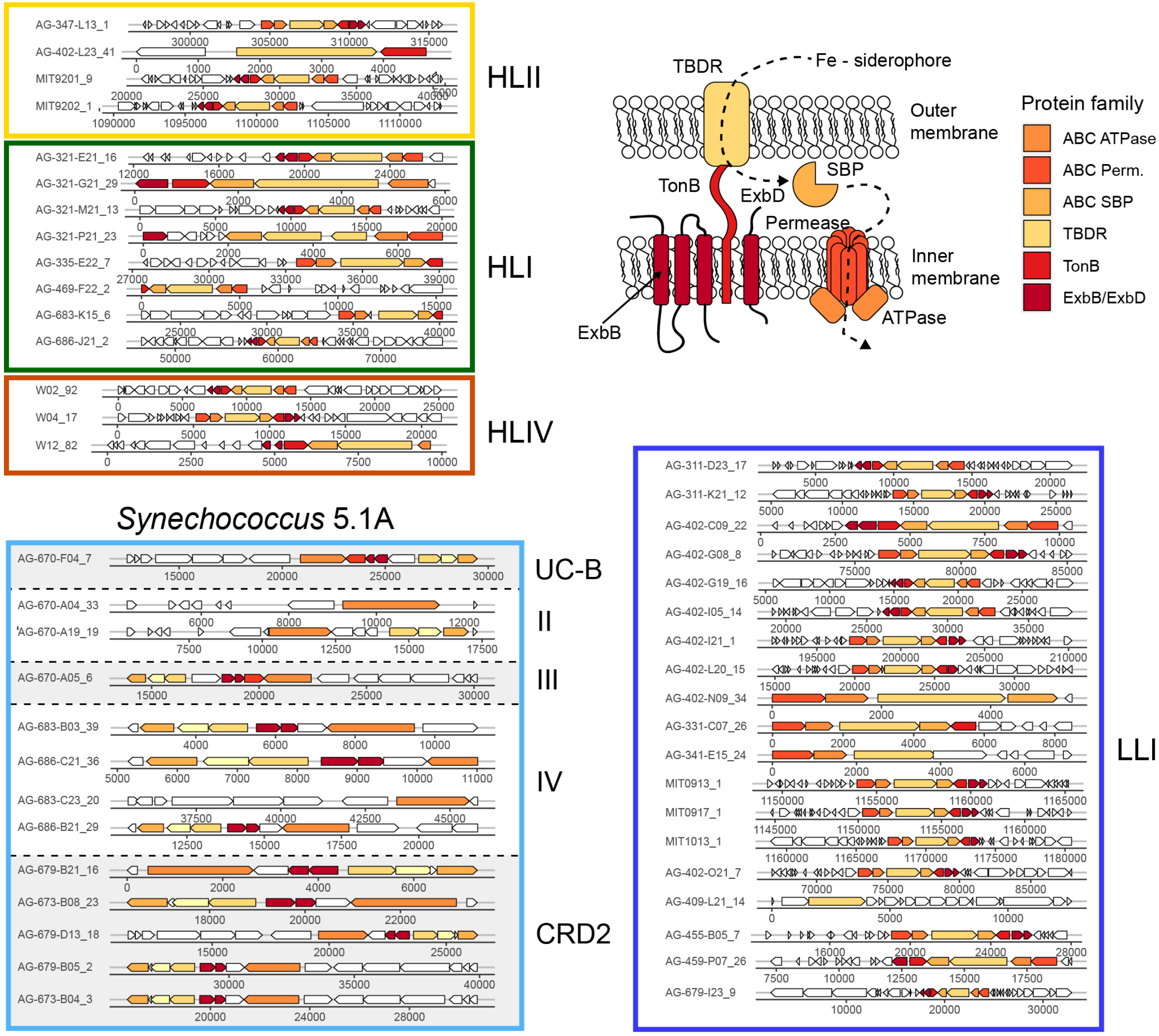

Supplementary Figure S1. Gene diagrams of siderophore transport clusters in marine picocyanobacteria Gram-negative bacteria import Fe-bound siderophores in a multistep process involving three protein modules: 1) an outer membrane, TonB-dependent receptor (TBDR) that recognizes and binds Fe-loaded siderophores, 2) an energy transducing complex (TonB/ExbB/ExbD) that translocates siderophores through the TBDR via proton motive force, and 3) an ATP-binding cassette (ABC) complex consisting of an inner membrane-bound permease/ATPase complex and a periplasmic solute binding protein [31]. Bacteria synthesize siderophores from secondary metabolite gene clusters encoding nonribosomal peptide synthetases and polyketide synthases [32]. The genes encoding both siderophore biosynthesis and uptake are typically clustered on the chromosome in an operon-like structure and are tightly regulated. Cartoon gene diagrams show the absolute position of siderophore transport components. Diagrams are partitioned by clade (colored/shaded boxes). The last number following each genome is the contig number of the gene cluster. ABC ATPase, ATP-binding cassette transporter ATPase component (COG1120); ABC Perm., ATP-binding cassette transporter membrane permease component (COG0609); ABC SBP, ATP-binding cassette transporter periplasmic solute binding component (COG0614); TBDR, outer membrane TonB-dependent receptor protein (COG4771); TonB, energy transducing protein TonB (COG0810); ExbB/ExbD, biopolymer transport proteins (COG0811).

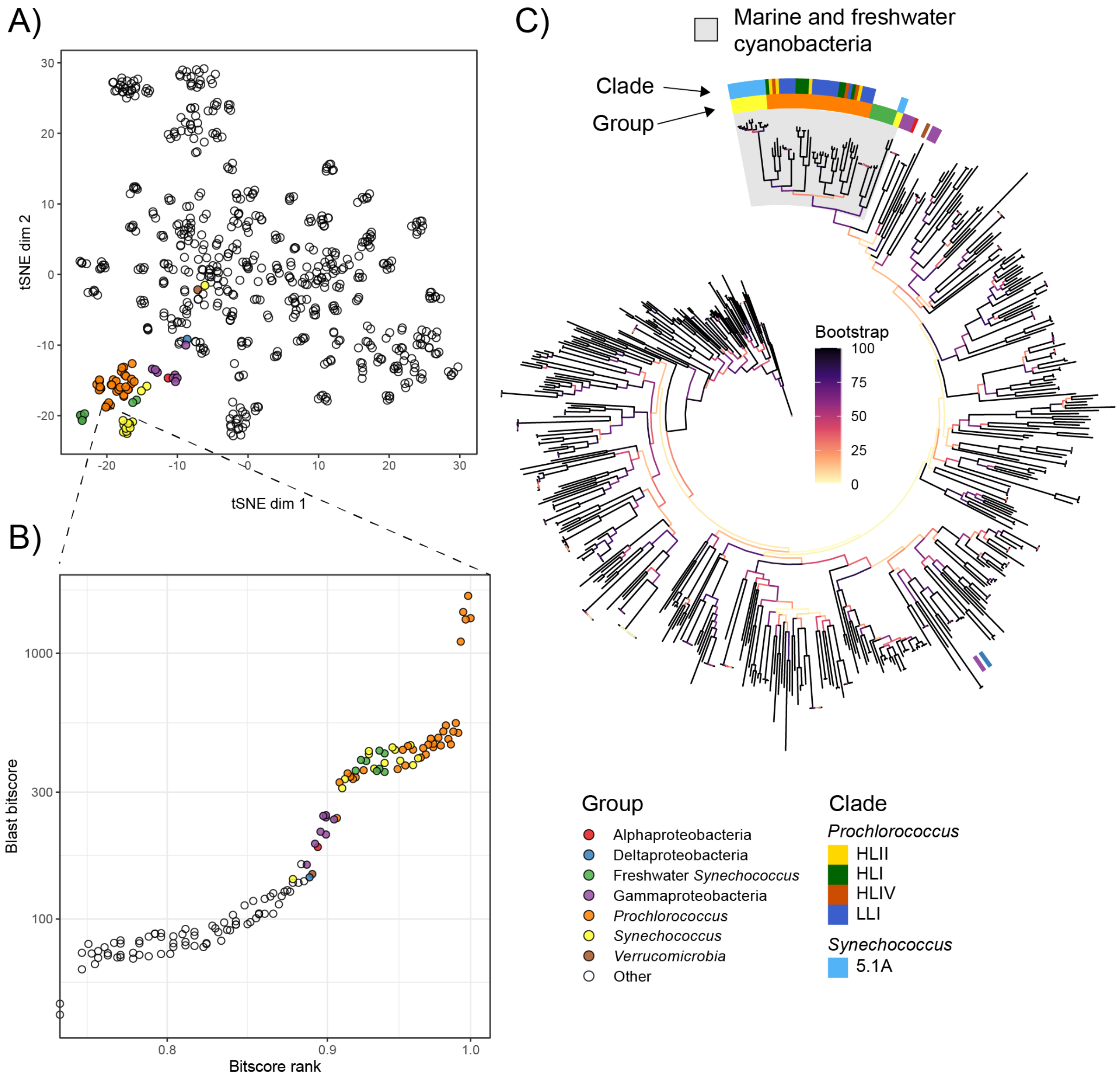

Supplementary Figure S2. Comparing the sequence similarity of picocyanobacterial TonB dependent receptor (TBDR) genes

A) t-Distributed Stochastic Neighbor Embedding (tSNE) dimensionality reduction of pairwise sequence similarity score between 625 TonB dependent receptor (TBDR) amino acid sequences with a pairwise blastp e value < 1e-10 to any marine picocyanobacterial TBDR. Sequences that share greater amino acid similarity cluster more closely together. The colored subset represents all sequences with an e value < 1e-40 to a marine picocyanobacterial TBDR, and they are colored by their taxonomic origin. **B)** Focused view of the sequence similarity scores of the TBDR from LLI *Prochlorococcus* strain MIT0917 and the most closely related TBDRs. Each point is a sequence comparison to the MIT0917 sequence, and the vertical axis shows the blastp bitscore of the comparison. The comparisons are bitscore rank-ordered (x-axis), showing only those from the top 25%. **C)** Unrooted maximum likelihood phylogenetic tree of the 625 TBDRs from A) and B). Branches are colored by bootstrap score out of 300 bootstraps. The inner ring highlights the same highlighted sequences from A) and B), and the outer ring categorizes sequences by picocyanobacterial clade.

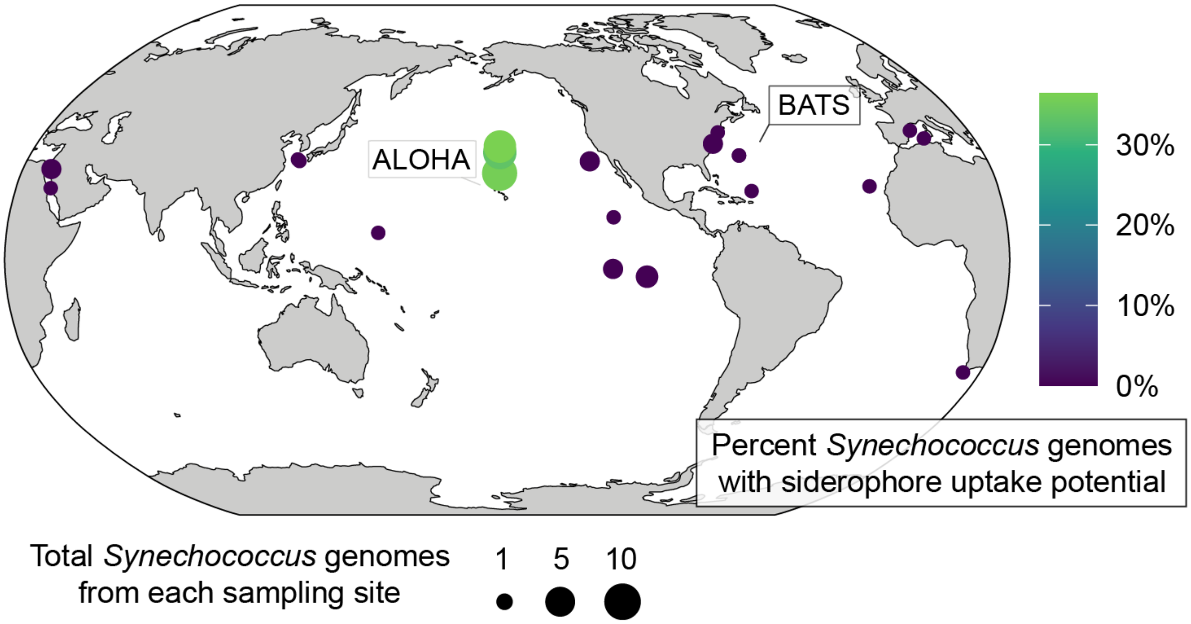

Supplementary Figure S3. Biogeography of *Synechococcus* siderophore consumers
Sampling locations of *Synechococcus* isolate and single-cell genomes used for this study. Point size is proportional to the total number of sampled genomes from each location, and point color is proportional to the percentage of genomes with siderophore uptake systems at that location. Oceanographic stations ALOHA and BATS are denoted for reference.

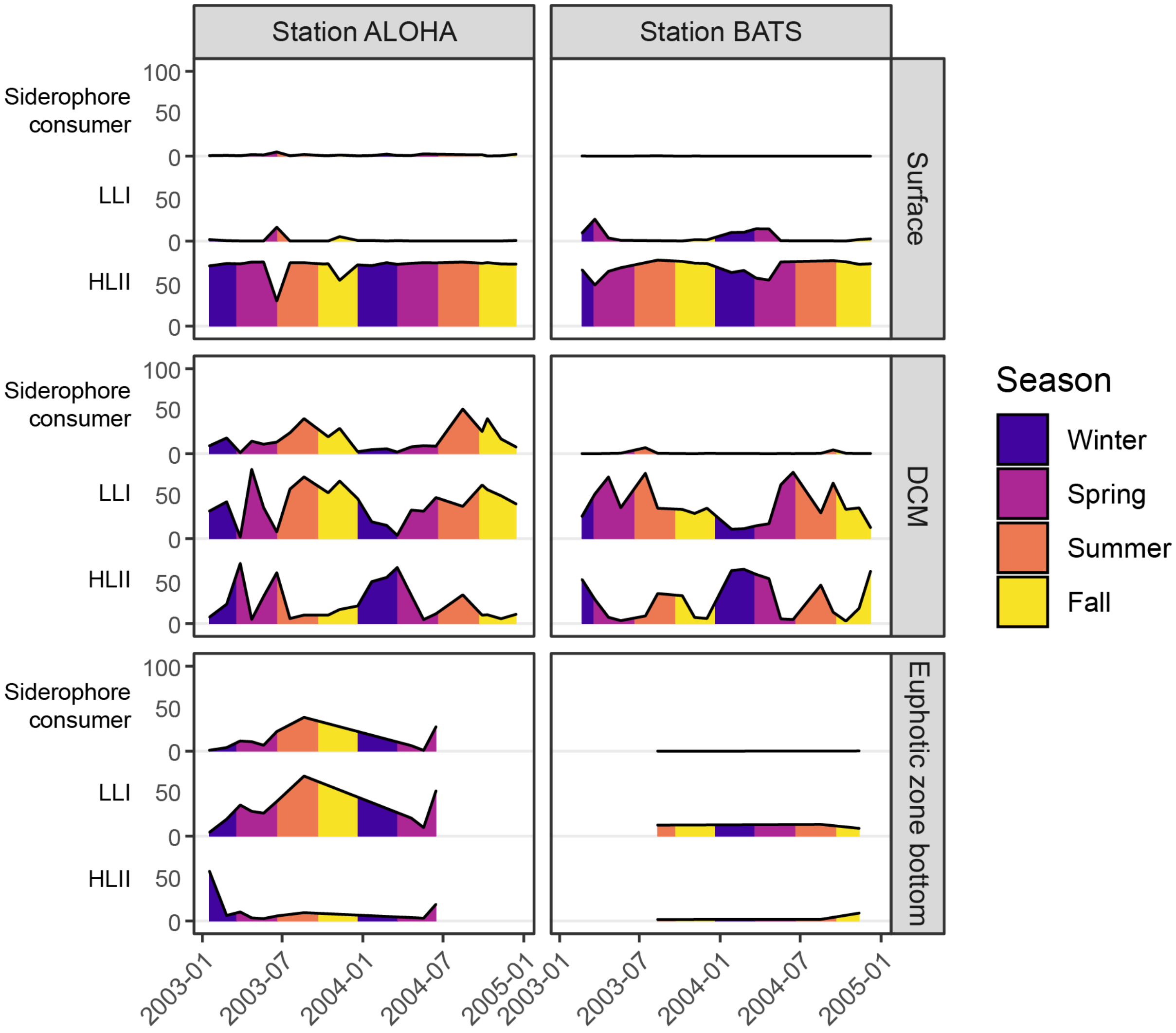

Supplementary Figure S4. Seasonal dynamics of *Prochlorococcus* siderophore consumers in the N. Pacific (Station ALOHA) and N. Atlantic (Station BATS)
Relative abundance (%) of *Prochlorococcus* siderophore consumers (vertical axis top), LLI clade (vertical axis middle), and HLII clade (vertical axis bottom) at the surface, DCM, and the bottom of the euphotic zone at HOT and BATS from 2003 to 2005. Area plots are colored by the season when samples were collected. The vertical axis shows the estimated percentage of all *Prochlorococcus* genomes with the siderophore uptake gene cluster or the estimated percentage of each clade out of the total *Prochlorococcus* community.

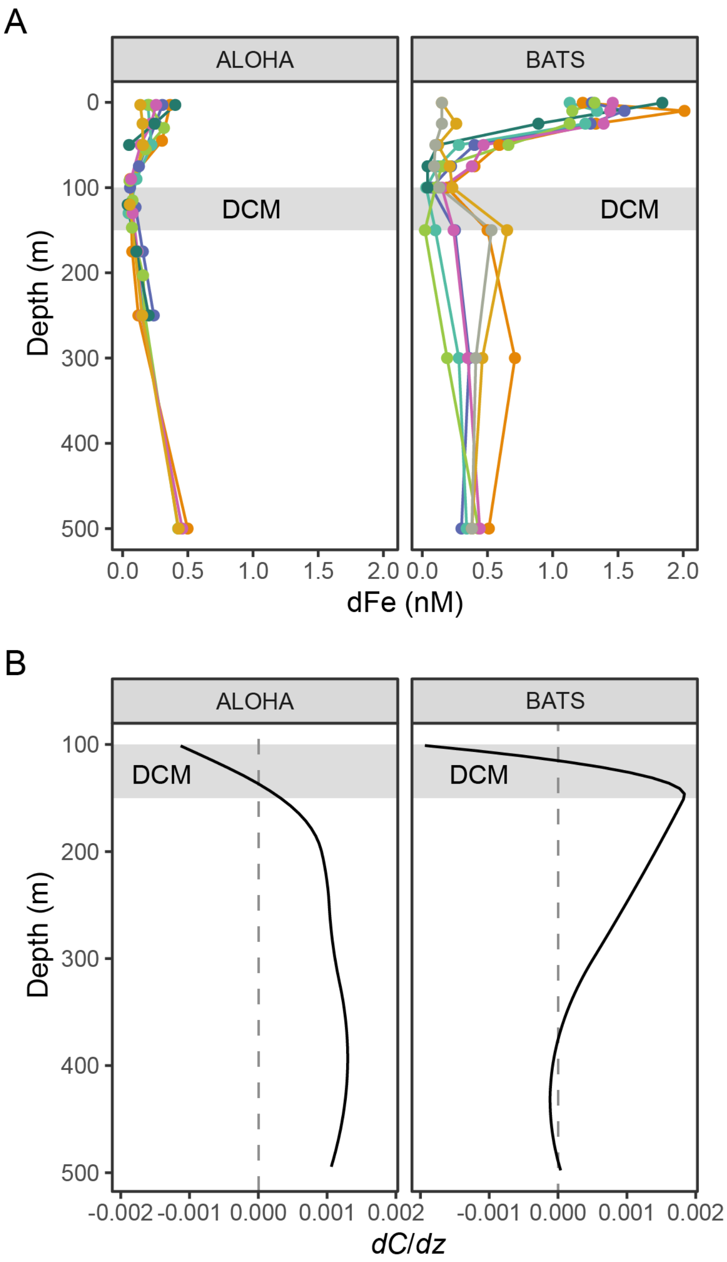

Supplementary Figure S5. dFe concentrations from the N. Pacific (Station ALOHA) and N. Atlantic (Station BATS) from prior studies.

A) Dissolved Fe (dFe) profiles from two prior studies at BATS [33] and ALOHA [34]. The DCM region is highlighted in grey. **B)** Change in dFe concentration with depth for the two locations. $\frac{dC}{dz}$ is estimated by taking the derivative of a thin plate smooth fit to dFe as a function of depth. Derivatives are estimated from Generalized Additive Models via finite differences. The magnitude of the rate of change is highest at the BATS DCM, which implies a larger relative flux of dFe from below the DCM than at ALOHA.

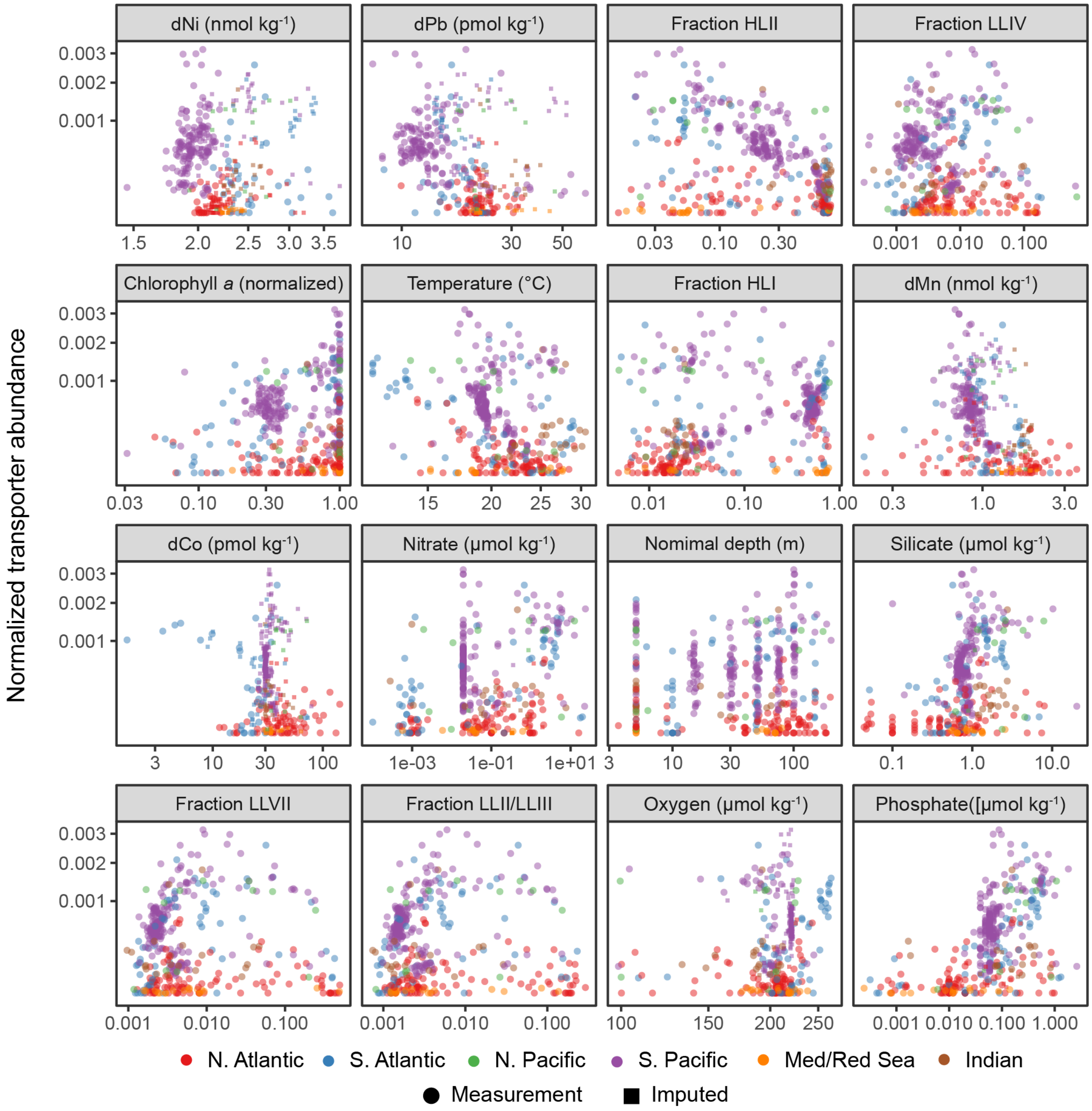

Supplementary Figure S6. Other predictive variables of *Prochlorococcus* siderophore consumer abundance Normalized abundance of *Prochlorococcus* siderophore receptors plotted against the remaining environmental variables with the greatest predictive contribution to the random forest model (Fig. 4). Each point is a metagenomic observation colored by ocean basin. Small squares represent samples with missing data that was imputed for each variable (see methods). Dissolved Fe climatological means are from the MIT Darwin model. The remaining measurements are *in situ* chemical, biological, or hydrographic measurements from GEOTRACES and Tara Oceans.

Supplementary Tables:

Supplementary Table S1. Regression of dFe, Fe-binding ligands, and siderophores at ALOHA and BATS

**A) Model summary** (GLM Gaussian)					
Measurement	Variable	Estimate	Std. Error	t value	p-value
dFe [nmol kg^{-1}]	(Intercept)	-1.0862	0.1959	-5.55	8.60e-07
dFe [nmol kg^{-1}]	$Ocean_{N. Pacific}$	-1.0749	0.2287	-4.70	1.78e-05
dFe [nmol kg^{-1}]	$Env_{Surface}$	0.2771	0.3158	0.88	0.3840
dFe [nmol kg^{-1}]	$ocean_{N. Pacific}$:$Env_{Surface}$	0.6793	0.3556	1.91	0.0613
AIC=103.592; BIC=113.980; R^2 = 0.55; RMSE=0.535; Dispersion = 0.306					
L_1 [nmol kg^{-1}]	(Intercept)	0.3031	0.1136	2.67	0.0110
L_1 [nmol kg^{-1}]	$Ocean_{N. Pacific}$	-1.0062	0.1444	-6.97	2.36e-08
L_1 [nmol kg^{-1}]	$Env_{Surface}$	-0.1243	0.1832	-0.68	0.5015
L_1 [nmol kg^{-1}]	$ocean_{N. Pacific}$:$Env_{Surface}$	0.8143	0.2181	3.73	0.0006
AIC=30.192; BIC=38.998; R^2 = 0.65; RMSE=0.306; Dispersion = 0.103					

**B) Model contrasts**						
Measurement	Ref. level	Contrast	Estimate	Std. Error	z-ratio	p-value
dFe [nmol kg^{-1}]	DCM	N. Pacific - N. Atlantic	-1.0749	0.2287	-4.700	<.0001
dFe [nmol kg^{-1}]	Surface	N. Pacific - N. Atlantic	-0.3956	0.2723	-1.453	0.5850
dFe [nmol kg^{-1}]	N. Atlantic	Surface - DCM	0.2771	0.3158	0.878	1.0000
dFe [nmol kg^{-1}]	N. Pacific	Surface - DCM	0.9564	0.1635	5.849	<.0001
L_1 [nmol kg^{-1}]	DCM	N. Pacific - N. Atlantic	-1.0062	0.1444	-6.969	<.0001
L_1 [nmol kg^{-1}]	Surface	N. Pacific - N. Atlantic	-0.1919	0.1635	-1.174	0.9615
L_1 [nmol kg^{-1}]	N. Atlantic	Surface - DCM	-0.1243	0.1832	-0.678	1.0000
L_1 [nmol kg^{-1}]	N. Pacific	Surface - DCM	0.6900	0.1184	5.829	<.0001

A) Regression summary for dFe and strong Fe-binding ligands (L_1) as a function of Ocean and depth (surface or DCM). Response error distributions was Gaussian with identity-link. **B)** Pairwise model contrasts. P value adjustment: Bonferroni method for 4 tests. Results are given on the log (not the response) scale.

Supplementary Table S2. Beta regression of *Prochlorococcus* siderophore receptor abundance

A) Model summary (mean model with logit link)

Coefficient	Estimate	Std. Error	z value	p-value
(Intercept)	-8.242708	0.139624	-59.035	< 2e-16
Nitrite $conc._{mid}$	0.713442	0.256566	2.781	0.00542
Nitrite $conc._{high}$	0.920409	0.157066	5.860	4.63e-09
DCM $Depth_{mid}$	-0.082504	0.177126	-0.466	0.64136
DCM $Depth_{high}$	0.005527	0.186588	0.030	0.97637
$Nitrite_{mid}$:DCM_{mid}	0.661713	0.304400	2.174	0.02972
$Nitrite_{high}$:DCM_{mid}	0.443413	0.237921	1.864	0.06236
$Nitrite_{mid}$:DCM_{high}	0.572505	0.304896	1.878	0.06042
$Nitrite_{high}$:DCM_{high}	0.734502	0.249463	2.944	0.00324

Pseudo $R^2 = 0.35$; Log Likelihood = 3296; Df = 10; Num. obs. = 407

B) Joint tests of the terms in the model

Model Term	Df1	Df2	F ratio	p-value
Nitrite conc.	2	Inf	71.83	<.0001
DCM Depth	2	Inf	11.81	<.0001
Nitrite conc:DCM Depth	4	Inf	5.58	0.0002

C) Model contrasts

DCM Depth	Contrast Nitrite conc.	Estimate	Std. Error	z ratio	p-value
mid	mid - low	7.15e-04	1.06e-04	6.75	8.80e-11
high	mid - low	6.91e-04	9.36e-05	7.39	7.96e-13

A) Beta regression summary for siderophore transporter relative abundance as function of NO_2 concentration [μmol kg^{-1}], DCM depth [m], and an interaction between the two terms. Response error distributions was Beta with logit-link. Estimated coefficients are on the logit scale. **B)** Interaction contrasts for all effects in the model compiled into a Type-III-ANOVA-like table. **C)** Pairwise model contrasts. For brevity only contrasts with $P \leq 0.05$ are included. P value adjustment: mvt method for 12 tests. Tests are performed on the logit scale.

References

1. Becker, J. W., Hogle, S. L., Rosendo, K. & Chisholm, S. W. Co-culture and biogeography of *Prochlorococcus* and SAR11. *ISME J.* **13,** 1506–1519 (2019).
2. Mukherjee, S. *et al.* 1,003 reference genomes of bacterial and archaeal isolates expand coverage of the tree of life. *Nat. Biotechnol.* **35,** 676–683 (2017).
3. Tully, B. J., Graham, E. D. & Heidelberg, J. F. The reconstruction of 2,631 draft metagenome-assembled genomes from the global oceans. *Sci Data* **5,** 170203 (2018).
4. Tully, B. J., Sachdeva, R., Graham, E. D. & Heidelberg, J. F. 290 metagenome-assembled genomes from the Mediterranean Sea: a resource for marine microbiology. *PeerJ* **5,** e3558 (2017).
5. Parks, D. H. *et al.* Recovery of nearly 8,000 metagenome-assembled genomes substantially expands the tree of life. *Nat Microbiol* (2017).
6. Mende, D. R. *et al.* proGenomes: a resource for consistent functional and taxonomic annotations of prokaryotic genomes. *Nucleic Acids Res.* **45,** 529–534 (2017).
7. Keeling, P. J. *et al.* The Marine Microbial Eukaryote Transcriptome Sequencing Project (MMETSP): illuminating the functional diversity of eukaryotic life in the oceans through transcriptome sequencing. *PLoS Biol.* **12,** e1001889 (2014).
8. Haroon, M. F., Thompson, L. R., Parks, D. H., Hugenholtz, P. & Stingl, U. A catalogue of 136 microbial draft genomes from Red Sea metagenomes. *Sci Data* **3,** 160050 (2016).
9. Hugerth, L. W. *et al.* Metagenome-assembled genomes uncover a global brackish microbiome. *Genome Biol.* **16,** 279 (2015).
10. Klemetsen, T. *et al.* The MAR databases: development and implementation of databases specific for marine metagenomics. *Nucleic Acids Res.* **46,** 692–699 (2018).
11. Berube, P. M. *et al.* Single cell genomes of Prochlorococcus, Synechococcus, and sympatric microbes from diverse marine environments. *Scientific Data* **5,** 180154 (2018).
12. Haft, D. H. *et al.* RefSeq: an update on prokaryotic genome annotation and curation. *Nucleic Acids Res.* **46,** 851–860 (2018).
13. Karsenti, E. *et al.* A holistic approach to marine eco-systems biology. *PLoS Biol.* **9,** e1001177 (2011).
14. Biller, S. J. *et al.* Marine microbial metagenomes sampled across space and time. *Scientific Data* **5,** 180176 (2018).
15. Malmstrom, R. R. *et al.* Temporal dynamics of *Prochlorococcus* ecotypes in the Atlantic and Pacific oceans. *ISME J.* **4,** 1252–1264 (2010).
16. Schlitzer, R. *et al.* The GEOTRACES Intermediate Data Product 2017. *Chem. Geol.* **493,** 210–223 (2018).
17. Salt, L. A., van Heuven, S. M. A. C., Claus, M. E., Jones, E. M. & de Baar, H. J. W. Rapid acidification of mode and intermediate waters in the southwestern Atlantic Ocean. *Biogeosciences* **12,** 1387–1401 (2015).
18. Rijkenberg, M. J. A. *et al.* The distribution of dissolved iron in the West Atlantic Ocean. *PLoS One* **9,** e101323 (2014).
19. Wyatt, N. J. *et al.* Biogeochemical cycling of dissolved zinc along the GEOTRACES South Atlantic transect GA10 at 40°S: Dissolved zinc in the Atlantic at 40º S. *Global Biogeochem. Cycles* **28,** 44–56 (2014).
20. Ashkezari, M. D. *et al.* Simons Collaborative Marine Atlas Project (Simons CMAP): An open-source portal to share, visualize, and analyze ocean data. *Limnol. Oceanogr. Methods* **19,** 488–496 (2021).

21. Dutkiewicz, S. *et al.* Capturing optically important constituents and properties in a marine biogeochemical and ecosystem model. *Biogeosciences* **12,** 4447–4481 (2015).

22. Ward, B. A., Dutkiewicz, S., Jahn, O. & Follows, M. J. A size-structured food-web model for the global ocean. *Limnol. Oceanogr.* **57,** 1877–1891 (2012).

23. Stekhoven, D. J. & Buhlmann, P. MissForest–non-parametric missing value imputation for mixed-type data. *Bioinformatics* **28,** 112–118 (2012).

24. Wright, M. & Ziegler, A. ranger: A Fast Implementation of Random Forests for High Dimensional Data in C++ and R. *Journal of Statistical Software, Articles* **77,** 1–17 (2017).

25. Stepanauskas, R. *et al.* Improved genome recovery and integrated cell-size analyses of individual uncultured microbial cells and viral particles. *Nat. Commun.* **8,** 84 (2017).

26. Bushnell, B., Rood, J. & Singer, E. BBMerge - Accurate paired shotgun read merging via overlap. *PLoS One* **12,** e0185056 (2017).

27. Menzel, P., Ng, K. L. & Krogh, A. Fast and sensitive taxonomic classification for metagenomics with Kaiju. *Nat. Commun.* **7,** 11257 (2016).

28. Camacho, C. *et al.* BLAST+: architecture and applications. *BMC Bioinformatics* **10,** 421 (2009).

29. Kursa, M. & Rudnicki, W. Feature Selection with the Boruta Package. *Journal of Statistical Software, Articles* **36,** 1–13 (2010).

30. Wood, S. N. *Generalized Additive Models: An Introduction with R* 2017.

31. Wandersman, C. & Delepelaire, P. Bacterial iron sources: from siderophores to hemophores. *Annu. Rev. Microbiol.* **58,** 611–647 (2004).

32. Crosa, J. H. & Walsh, C. T. Genetics and assembly line enzymology of siderophore biosynthesis in bacteria. *Microbiol. Mol. Biol. Rev.* **66,** 223–249 (2002).

33. Sedwick, P. N. *et al.* Iron in the Sargasso Sea (Bermuda Atlantic Time-series Study region) during summer: Eolian imprint, spatiotemporal variability, and ecological implications. *Global Biogeochem. Cycles* **19** (2005).

34. Fitzsimmons, J. N. *et al.* Daily to decadal variability of size-fractionated iron and iron-binding ligands at the Hawaii Ocean Time-series Station ALOHA. *Geochim. Cosmochim. Acta* **171,** 303–324 (2015).
